# Antioxidant Activity of Hawaiian Marine Algae

**DOI:** 10.3390/md10020403

**Published:** 2012-02-15

**Authors:** Dovi Kelman, Ellen Kromkowski Posner, Karla J. McDermid, Nicole K. Tabandera, Patrick R. Wright, Anthony D. Wright

**Affiliations:** 1 Department of Pharmaceutical Sciences, College of Pharmacy, University of Hawaii at Hilo, 34 Rainbow Drive, Hilo, HI 96720, USA; Email: dkelman@hawaii.edu (D.K.); ntabande@hawaii.edu (N.K.T.); patrickrw@gmx.net (P.R.W.); 2 Department of Marine Science, University of Hawaii at Hilo, 200 West Kawili Street, Hilo, HI 96720, USA; Email: ellenr@hawaii.edu (E.K.P.); mcdermid@hawaii.edu (K.J.M.); 3 Summer Scholar visiting from University of Freiburg, Freiburg 79085, Germany

**Keywords:** antioxidant activity, algae, Hawaii, *Turbinaria*, carotenoids, fucoxanthin, chemoprevention

## Abstract

Marine algae are known to contain a wide variety of bioactive compounds, many of which have commercial applications in pharmaceutical, medical, cosmetic, nutraceutical, food and agricultural industries. Natural antioxidants, found in many algae, are important bioactive compounds that play an important role against various diseases and ageing processes through protection of cells from oxidative damage. In this respect, relatively little is known about the bioactivity of Hawaiian algae that could be a potential natural source of such antioxidants. The total antioxidant activity of organic extracts of 37 algal samples, comprising of 30 species of Hawaiian algae from 27 different genera was determined. The activity was determined by employing the FRAP (Ferric Reducing Antioxidant Power) assays. Of the algae tested, the extract of *Turbinaria ornata* was found to be the most active. Bioassay-guided fractionation of this extract led to the isolation of a variety of different carotenoids as the active principles. The major bioactive antioxidant compound was identified as the carotenoid fucoxanthin. These results show, for the first time, that numerous Hawaiian algae exhibit significant antioxidant activity, a property that could lead to their application in one of many useful healthcare or related products as well as in chemoprevention of a variety of diseases including cancer.

## 1. Introduction

Marine algae produce a diverse array of compounds that function as chemical defense systems facilitating their survival in extremely competitive environments [1]. Research into the natural products chemistry and chemical defenses of algae over the past 40 years has resulted in the isolation of over 15,000 novel compounds, many of which have been shown to have bioactive properties [1,2,3]. Algae from the three groupings traditionally known as Chlorophyta (green algae), Rhodophyta (red algae), and Phaeophyta (brown algae) produce compounds with varying bioactivities [4]. In light of the broad spectrum of their reported biological activities, algae have been suggested as a promising source of bioactive substances that might have pharmaceutical applications [4].

Marine algae in shallow water habitats can be exposed to a combination of ultraviolet light and air that readily leads to the formation of free radicals and other reactive oxygen species (ROS). Despite their exposure to harmful ROS, healthy algae lack oxidative damage in their structural components (*i.e.*, fatty acids) and resist oxidation during storage, indicating the presence of protective antioxidant defense systems in their cells [5,6]. By donating an electron, antioxidants neutralize free radicals that would otherwise oxidize biomolecules leading to cell death and tissue damage [6,7,8]. Accordingly, interest in the search for natural antioxidants from algae has been increasing in recent years. The overall aim of this type of research is discovery of compounds and/or extracts that can counteract free radical-induced and other oxidative stress processes, and in so doing decrease the incidence of human diseases directly related to these processes [9].

Antioxidant activity has been reported in numerous genera of marine algae, including *Ahnfeltiopsis*, *Colpomenia*, *Gracilaria*, *Halymenia*, *Hydroclathrus*, *Laurencia*, *Padina*, *Polysiphonia*, and *Turbinaria* [10]. Natural antioxidants from algae are known to play an important role against various diseases and aging processes [11]. The detected antioxidant compounds in algae from these genera and others have potential anti-aging, dietary, anti-inflammatory, antibacterial, antifungal, cytotoxic, anti-malarial, anti-proliferative, and anticancer properties [10,11]. Although there are publications on the antioxidant activity of numerous genera of algae, many of which are commonly found in the Hawaiian Islands, there are no reports of systematic testing of Hawaiian species of algae for their antioxidant activity.

In the Hawaiian Islands there are approximately 520 reported species of marine algae [12,13], very few of which have been investigated biochemically in any way. In one of the few systematic studies, McDermid and Stuercke [14] reported on the nutritional composition of 22 species of Hawaiian algae, testing for protein, lipid, carbohydrate, ash, caloric, mineral, and vitamin content. The tropical brown alga, *Dictyota acutiloba*, from Kauai Island was shown to contain a compound with potent antiherbivore activity [15]. Only one study has been previously published on the antioxidant activity of Hawaiian algae: Vijayavel and Martinez [9] found that extracts of *Ulva fasciata* and *Gracilaria salicornia* from Oahu Island exhibited significant antioxidant activity. These algal extracts also showed antimicrobial activity. Beyond this finding, there is no published information on the antioxidant activity of Hawaiian algae. It is expected that additional Hawaiian algae contain very effective antioxidant systems, as they are exposed to prolonged intense ultraviolet (UV) radiation in their tropical environment. In fact, it has been observed that UV radiation stimulates antioxidant defense in algae [11].

In light of the potential commercial uses of algal antioxidant compounds in the medicine, food, pharmaceutical, and cosmetic industries [11], we saw the need to ascertain whether Hawaiian algae could be a natural source of such compounds. Therefore, the present study was conducted to quantify the antioxidant activity of extracts from 37 samples of algae, comprising 30 species of Hawaiian algae from 27 different genera (Table 1) using the ferric reducing antioxidant power (FRAP) assay. Furthermore, we used bioassay-guided fractionation to isolate and identify the major antioxidant compound from the species of algae whose extract was found to have the highest activity.

**Table 1:** Collection location, date and depth of Hawaiian algae analyzed for antioxidant activity.

Species	Collection Location, Island	Collection Date (day/month/year)	Depth (m)	Sample Number
**Phaeophyta**				
*Colpomenia sinuosa*	Leleiwi Beach Park, Hawaii	7, November, 2009	−0.5	A0014
*Dictyopteris plagiogramma*	Makai Pier, Oahu	5, August, 2003	−3.0	A0023
*Hydroclathrus clathratus*	Leleiwi Beach Park, Hawaii	7, November, 2009	−1.5	A0016
*Sargassum echinocarpum*	Richardson’s Ocean Park, Hawaii	6, September, 2004	−0.5	A0030
*Sargassum obtusifolium*	Richardson’s Ocean Park, Hawaii	6, September, 2004	−0.5	A0033
*Spatoglossum macrodontum*	Penguin Bank, off Molokai	17 September, 2004	−78.0	A0021
*Spatoglossum macrodontum*	Penguin Bank, off Molokai	March 2009	−81.0	A0042
*Turbinaria ornata*	Richardson’s Ocean Park, Hawaii	17 August, 2004	−0.5	A0031
*Turbinaria ornata*	Waikoloa, Hawaii	12, March, 2011	−0.5	A0050
**Chlorophyta**				
*Chaetomorpha antennina*	Richardson’s Ocean Park, Hawaii	26, October, 2009	0.25	A0003
*Chaetomorpha antennina*	Punaluu Beach Park, Hawaii	15, July, 2004	0.25	A0029
*Chlorodesmis caespitosa*	Onekahakaha Beach Park, Hawaii	29 April, 2010	−1.0	A0038
*Codium mamillosum*	Penguin Bank, off Molokai	22, September, 2004	−78.0	A0020
*Derbesia tenuissima*	Fish tank, Marine Science Building	1, March, 2010	−0.5	A0036
*Gayralia oxysperma*	Richardson’s Ocean Park, Hawaii	26, October, 2009	0.1	A0004
*Gayralia oxysperma*	Richardson’s Ocean Park, Hawaii	6 September, 2004	0.1	A0034
*Rhizoclonium africanum*	Leleiwi Beach Park, Hawaii	7, November, 2009	0.1	A0013
*Ulva* sp.	Penguin Bank, off Molokai	26, March, 2009	−104	A0041
**Rhodophyta**				
*Acanthophora pacifica*	Punaluu Beach Park, Hawaii	15, July, 2004	−2.0	A0027
*Acanthophora spicifera*	Richardson’s Ocean Park, Hawaii	26, October, 2009	−0.5	A0002
*Ahnfeltiopsis concinna*	Four-mile Beach Park, Hawaii	2, November, 2009	0.2	A0005
*Ahnfeltiopsis concinna*	King’s Landing, Hawaii	7, November, 2009	0.2	A0007
*Ahnfeltiopsis concinna*	Richardson’s Ocean Park, Hawaii	26, October, 2009	0.2	A0001
*Amansia glomerata*	Kapoho, Hawaii	9, November, 2009	−1.0	A0018
*Chondrus ocellatus*	Leleiwi Beach Park, Hawaii	7, November, 2009	−0.5	A0011
*Halymenia formosa*	Mahaiula Bay, Hawaii	1, August, 2003	−2.5	A0028
*Hypnea spinella*	King’s Landing, Hawaii	7, November, 2009	0.1	A0008
**Rhodophyta**				
*Laurencia galtsoffii*	Kapoho, Hawaii	10, November, 2009	−2.0	A0019
*Laurencia mcdermidiae*	Makapuu, Oahu	5, August, 2003	0.1	A0022
*Peyssonnelia inamoena*	Penguin Bank, off Molokai	25, March, 2009	−109	A0039
*Polyopes hakalauensis*	King’s Landing, Hawaii	7, November, 2009	0.1	A0009
*Polysiphonia howei*	Leleiwi Beach Park, Hawaii	7, November, 2009	0.1	A0012
*Portieria hornemanni*	Richardson’s Ocean Park, Hawaii	2, February, 2010	−1.0	A0037
*Pterocladiella capillacea*	Four-mile Beach Park, Hawaii	2, November, 2009	0.0	A0006
*Pterocladiella capillacea*	King’s Landing, Hawaii	7, November, 2009	0.0	A0010
*Stenopeltis gracilis*	Leleiwi Beach Park, Hawaii	7, November, 2009	−1.0	A0015
*Tricleocarpa cylindrica*	Richardson’s Ocean Park, Hawaii	6, September, 2004	−1.0	A0032

## 2. Results

**Figure 1:**
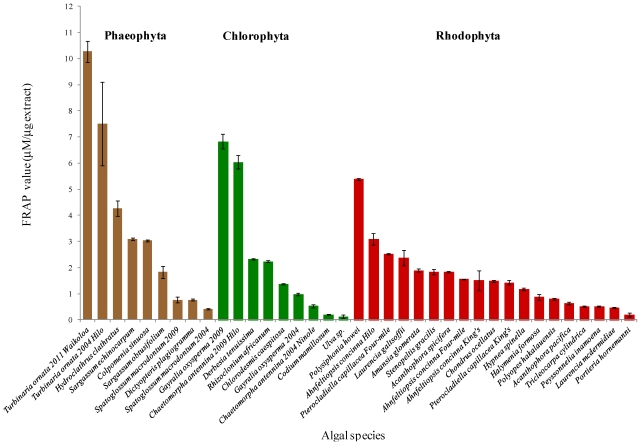
Total antioxidant activity of organic extracts of Hawaiian algae presented as mean FRAP values in μM per μg extract. Error bars ± SD. The different colors represent the different algal groups. In cases where there are more than one sample of the same species collected at a different date or location (see Table 1), the date and/or location code was added. Hilo for Richardson’s Ocean Park; Ninole,for Punaluu Beach Park; Four-mile for Four-mile Beach Park; King’s, for King’s Landing.

Different Hawaiian algae had widely varying antioxidant activities, ranging from 0.13 ± 0.07 µM/µg extract for *Ulva* sp., from Penguin Bank, off Molokai Island, to 10.27 ± 0.40 µM/µg extract for *Turbinaria ornata* from Waikoloa, Hawaii Island (Figure 1).

A one-way ANOVA test revealed that the antioxidant activity varied significantly among algae (*P* << 0.001). The brown algae as a group had the highest mean antioxidant activity among Hawaiian algae with a mean FRAP value of 3.55 ± 3.16 µM/µg extract, followed by the green algae with a mean FRAP value of 2.29 ± 2.34 µM/µg extract. The red algae assessed had the lowest mean antioxidant activity with a FRAP value of 1.59 ± 1.17 µM/µg extract. A one-way analysis of variance comparing the antioxidant activity of the different algal groups revealed that the differences in antioxidant activity of the brown, green, and red algae were statistically significant (*P* < 0.01). However, *post hoc* comparisons indicated that the significance in the antioxidant activity of the different algal groups was due to the difference between the brown and red algae (*P* < 0.001). However, there was no statistical difference between the antioxidant activity of the brown and green algae (*P* = 0.067), nor between the green and red algae (*P* = 0.283).

Although differences in antioxidant activities were found among algae collected during different years, there was no significant correlation between samples’ ages (*i.e.*, the length of time between collection and analysis) and their antioxidant activities (*R*^2^ = 0.059).

A comparison between the antioxidant activities of algae collected from different depths revealed that those from shallow waters (0–3 m) were more active than those from depths in excess of 70 m (one-way ANOVA, *P* < 0.001), with mean FRAP values of 2.26 ± 2.29 and 0.21 ± 0.13 µM/µg extract, respectively.

Both *Turbinaria ornata* brown algal samples collected from Waikoloa in 2011 and Richardson’s Ocean Park, near Hilo, in 2004 showed the highest antioxidant activity among all tested samples; with FRAP values of 10.27 ± 0.40 and 7.50 ± 1.59 µM/µg extract, respectively (Figure 1). The next most active samples were of the green algae *Gayralia oxysperma* and *Chaetomorpha antennina*, both collected from Richardson’s Ocean Park in 2009, followed by the red alga *Polysiphonia howei*; with FRAP values of 6.82 ± 0.28, 6.04 ± 0.25 and 5.39 ± 0.03 µM/µg extract, respectively (Figure 1).

**Figure 2:**
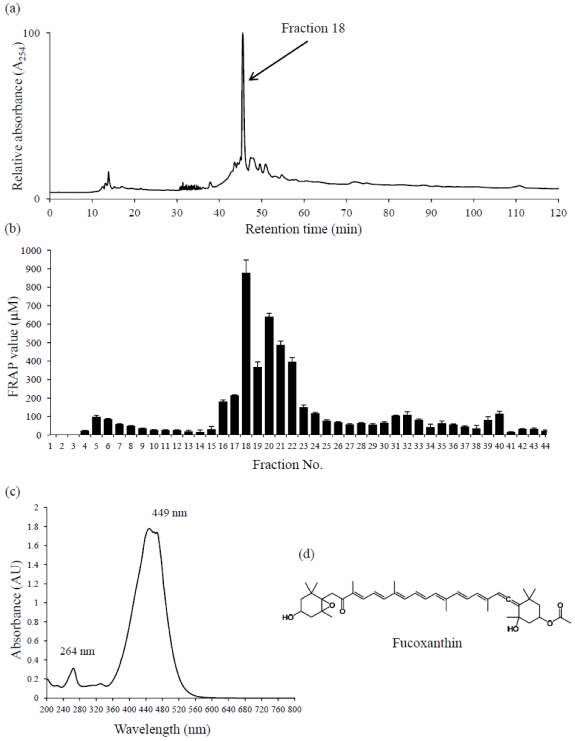
Bioassay-guided fractionation of *Turbinaria ornata* and the isolation of fucoxanthin as the active antioxidant: (**a**) Preperative HPLC chromatogram of *T. ornata* methanol extract; relative absorbance at 254 nm is presented. The peak that correlates to fraction 18 is indicated; (**b**) Total antioxidant activity of fractions collected from the preperative HPLC of *T. ornata* extract, presented as mean FRAP value in µM. Error bars ± SD; (**c**) The UV-visible absorbance spectrum corresponding to the HPLC peak of purified fraction 18. λ_max_ at 264 and 449 nm are indicated; and, (**d**) Chemical structure of fucoxanthin, the major antioxidant component of *T. ornata* extract.

In an attempt to determine the component(s) responsible for the activity of the most active extract, *Turbinaria ornata*, collected from Waikoloa in 2011, was chosen for bioassay-guided fractionation. HPLC fractionation of the active extract (Figure 2a) yielded a number of fractions with varying antioxidant activities (Figure 2b), fraction 18 being found to be the most active. This fraction was further purified (>98%) and analyzed by ^1^H and ^13^C NMR and ESI-MS ([M + Na]^+^ of 682 *m/z*), which showed it to be a carotenoid with a molecular weight of 659 amu. The only known carotenoid with this molecular weight is fucoxanthin. Further examination of our spectroscopic data, molecular weight, structural elements determined from ^1^H and ^13^C NMR, and its UV-Vis spectrum (Figure 2c), as well as comparisons made with an authentic sample confirmed the compound to indeed be fucoxanthin (Figure 2d).

## 3. Discussion

### 3.1. Phaeophyta

*Turbinaria ornata* samples collected in 2004 from Richardson’s Ocean Park, near Hilo, and in 2011 from Waikoloa (both on the island of Hawaii), exhibited the highest antioxidant activity of all the Hawaiian algae tested in this study (Figure 1). *Turbinaria conoides* and *T. ornata* from India have also been shown to possess significant antioxidant activity [16,17]. Crude and purified sulfated polysaccharides from *T. ornata* and *T. conoides*, respectively, have been shown to be excellent antioxidants, and have been suggested as natural antioxidant agents for use in the food industry or in the management of oxidative stress [17,18]. In recent years, sulfated polysaccharides have been shown to possess a wide range of bioactive properties, including antiviral, anticancer, anti-inflammatory, anticoagulant, and antioxidant activity [18]. The sulfated polysaccharides responsible for *T. conoides*’ antioxidant activity have been isolated and identified as fucoidan, laminaran, and alginate [18]. In the present study, the major antioxidant isolated and identified from the Hawaiian specimen of *T. ornata* was shown to be the carotenoid fucoxanthin (Figure 2).

More than 750 different carotenoids have been identified from nature [19], where fucoxanthin is structurally unique due to the presence of the somewhat unusual allenic moiety (C=C=C) [19,20]. Nevertheless, fucoxanthin is one of the most abundant carotenoids known and with remarkable biological activities [20]. In a very recent review [20], the biological activities and metabolism of fucoxanthin were described in detail. This unique carotenoid was found to possess potent anti-inflammatory, anticancer, anti-obese, anti-diabetic, anti-angiogenic, anti-malarial, and antioxidant activities, as well as protective effects on the liver, blood vessels of the brain, bones, skin, and eyes, including chemoprevention of a variety of diseases including cancer [20,21,22,23,24,25,26,27]. Fucoxanthin was shown to have a much higher antioxidant activity than other antioxidants, including tocopherols, and this higher activity is assumed to be due to the presence of the allenic moiety [26]. Fucoxanthin has been isolated primarily from brown algae and diatoms [19,20]. From brown algae, fucoxanthin has been isolated from 14 different genera, including *Alaria*, *Cladosiphon*, *Cystoseira*, *Eisenia*, *Fucus*, *Hijikia*, *Ishige*, *Kjellmaniella*, *Laminaria*, *Myagropsis*, *Padina*, *Petalonia*, *Sargassum*, and *Undaria* [20]. Our present study is the first report on the isolation of fucoxanthin from *Turbinaria* *ornata*, and could prove as a new resource of this important carotenoid.

The Hawaiian brown macroalgae, *Hydroclathrus clathratus*, *Sargassum echinocarpum*, and *Colpomenia sinuosa* also exhibited relatively high antioxidant activity (Figure 1). Heo *et al.* [28] found that *H. clathratus* and *C. sinuosa* from Jeju Island, Korea possessed similar antioxidant activity. However, our results could not be directly compared with these data due to differences in units and assay methods. In another study, extracts of *H. clathratus* inhibited the growth of human and monkey cancer cells, suppressed tumors, and demonstrated antioxidant activity [29]. Algae of the genus *Sargassum* have been well-studied, and extracts of many species shown to have high antioxidant activity [11]. Kang *el al*. [30] have shown that polyphenols are the major antioxidants in several species of Korean brown algae. The current study presents the first screening of the antioxidant activity of *S. echinocarpum*, a species endemic to Hawaii with significant cultural importance. Although the compounds responsible for the observed antioxidant activity are still to be isolated, it is not unlikely that its extract could have pharmaceutical and medicinal applications, as already evidenced by extracts of non-Hawaiian species being reported to ameliorate oxidative stress and reverse endothelial dysfunction in diabetic rats [31].

### 3.2. Chlorophyta

*Gayralia oxysperma* and *Chaetomorpha antennina* demonstrated the highest level of antioxidant activity of the tested Hawaiian green algae tested (Figure 1). This is the first report of the antioxidant activity of *G. oxysperma*, an alga that is consumed in Japan and known as *aonori*. *Gayralia nitidum* (formerly *Monostroma nitidum*) from Jeju Island, Korea has been reported to show antioxidant activity [28]. The level of antioxidant activity of *G. oxysperma* reported here, combined with its known antiviral and anticoagulant activities [32,33], as well as its high caloric value [14], makes it a potential valuable natural resource for the pharmaceutical, medicine, food, and aquaculture industries worthy of further research.

The significant antioxidant activity of *Chaetomorpha antennina* reported in the current study is in agreement with that reported by Premalatha and colleagues [34]. Their screening of extracts of this species revealed the presence of antioxidant compounds such as phenolics, tannins, and glycosides. In light of its antioxidant activity it is another local species that is being considered for further investigation.

The large disparity in antioxidant activity between the different samples of *Gayralia oxysperma* and *Chaetomorpha antennina* may be attributed to their different collection sites and/or dates. The samples of *G. oxysperma* were collected from the same site during different years, while the *C. antennina* samples were collected from different sites during different years (Table 1, Figure 1). For both species, the more recent samples exhibited higher antioxidant activity than the older ones. The fact that the samples with the lowest antioxidant activity were five years older than the more active samples could indicate long term storage as an important variable to be considered when measuring antioxidant activity. The deduction is not too surprising as loss of antioxidant activity for some frozen vegetables over eight months of storage has been reported [35]. Clearly, further research is needed in this area to determine the effects of long-term storage on the bioactivity and antioxidant activity of frozen algal samples.

The sample of *Chaetomorpha antennina* with the higher antioxidant activity was collected from Richardson’s Ocean Park on the eastern, windward coast of Hawaii Island near Hilo, while the less active sample was collected from Punaluu Beach Park on the southern shore near Ninole (Figure 1). The observed variation in antioxidant activity for these two samples could be due to slight differences in prevailing extrinsic factors, such as herbivory, light levels, depth, salinity, and nutrient levels [11]. Since these samples were also collected in different years, and different times of year, it is possible that the algae were in different growth or reproductive stages, variables known to affect compound levels in various algal species [11].

### 3.3. Rhodophyta

Of the red algae tested, *Polysiphonia howei* exhibited the highest antioxidant activity (Figure 1). Other studies on *P. urceolata* and *P. morrowii* have shown their extracts to have antioxidant activity [11,36]. In the current study, *P. howei* showed higher antioxidant activity than all tested species of *Laurencia* from the same order, Ceramiales. This observation is in accordance with previous studies, in which *Laurencia* spp., although a good source of biologically active secondary metabolites, do not exhibit high antioxidant activity [11]. Considering the broad spectrum of bioactivity of *Polysiphonia* extracts [10], it should be considered a promising source of not only natural antioxidants, but also a wide range of bioactive compounds.

Extracts of samples of *Ahnfeltiopsis concinna* and *Pterocladiella capillacea*, two of the most common intertidal red algae in the Hawaiian Islands, also showed relatively high antioxidant activity among the tested red algae (Figure 1). Neither species has been tested previously for antioxidant activity, even though extracts of *A. flabelliformis* have been found to exhibit antioxidant activity [37]. *A. devoniensis* extracts found to contain mycosporine-like amino-acids were also shown to have antioxidant activity [38]. The antioxidant activity of *A. concinna* is especially relevant in the Hawaiian Islands where it is known as *limu ‘aki’aki*, and eaten raw with limpets or baked with other foods [39].

The differences in antioxidant activity for the samples of *Ahnfeltiopsis concinna* (*A. concinna*) are probably attributable to the samples’ originating from different collection sites. The sample showing the highest antioxidant activity was collected from Richardson’s Ocean Park, while the other two were collected from Four-mile Beach Park and Leleiwi Beach Park. Each sample was collected in 2009. While all three samples were collected from high intertidal regions, it is possible that the *A. concinna* from Richardson’s Ocean Park was growing in a more stressful environment than those of the other samples, and consequently produced more antioxidant compounds in response to factors such as high light levels, large temperature fluctuations, and desiccation.

In a general sense, this study has demonstrated the potential of Hawaiian algae as sources of useful antioxidants and shown the need for further and more detailed studies in this area.

## 4. Experimental Section

### 4.1. Macroalgae Collection

During 2003, 2004, and 2009–2011, marine algae were collected in approximate 0.5–1 kg wet weight quantities from various locations around the islands of Hawaii, Oahu, and on Penguin Bank, off Molokai (Table 1), and transported in cooled insulated containers to our laboratory at the University of Hawaii at Hilo. Species from the groupings traditionally known as Chlorophyta, Phaeophyta, and Rhodophyta were selected for collection based on their potential to contain bioactive compounds, as indicated by the results of past research of related species in the same genera, such as *Laurencia*, *Sargassum*, *Codium*, *Chondrus*, *Porphyra*, *Portieria*, *Acanthophora*, *Ahnfeltiopsis*, *Caulerpa*, *Ulva*, and *Lobophora*. Algal samples were identified to genus and species levels when possible based on their morphology and anatomy, and by using taxonomic references [12,13,39]. Voucher specimens were prepared as dried specimens or slides and deposited in the Bishop Museum Herbarium in Honolulu (BISH).

### 4.2. Sample Preparation

Within six hours of collection, fresh algae were thoroughly rinsed three times with filtered seawater. Samples were then divided into 50–200 g portions, spun in a salad spinner for 30 s, weighed, frozen and then freeze-dried. Dry samples were weighed and the weight recorded.

### 4.3. Extractions

Samples were extracted with methanol (2 × 150 mL), first for approximately two hours and then for a further 24 h with occasional mixing at room temperature (25 °C). Resultant extracts were filtered and solvent removed under reduced pressure to yield dry material. Extracts’ weights were recorded. For the antioxidant activity assays a sample of each extract was prepared to give a final test concentration of 10 mg/mL in DMSO. All samples were stored at 4 °C until used.

### 4.4. Antioxidant Activity Assay

Antioxidant activity of algal extracts was determined using the ferric reducing antioxidant power (FRAP) assay, modified from the Benzie and Strain protocol [40,41]. The working FRAP reagent was made by mixing 300 mM acetate buffer (pH 3.6), 10 mM 2,4,6-tripyridyl-*S*-triazine (TPTZ) solution, and 20 mM FeCl_3_·6H_2_O in a 10:1:1 ratio and heated to 37 °C prior to use. The 300 mM acetate buffer was prepared by mixing 3.1 g of sodium acetate trihydrate (NaOAc·3H_2_O) with 16 mL glacial acetic acid and made to 1 L with ddH_2_O. The TPTZ solution was prepared by mixing equal volumes of 10 mM TPTZ with 40 mM HCl.

For the actual assays, 150 µL of FRAP reagent was added to each well of a 96-well microtiter plate. A blank reading was taken at 595 nm using a Bio-Rad (Hercules, CA, USA) microtiter plate reader. To each well 20 µL of sample in triplicate was then added, incubated for 8 min at room temperature and read at 595 nm. Triplicate standards of known Fe^II^ concentrations were run simultaneously using concentrations between 50 and 1000 µM of FeSO_4_·7H_2_O. A standard curve was plotted and FRAP values, in µM, determined. Since results may vary between plates, a new standard curve was prepared for each plate.

### 4.5. Bioassay-Guided Fractionation, Isolation, and Identification of the Active Principle

Preliminary investigations indicated that the extract of *Turbinaria ornata* had the highest antioxidant activity among the algae investigated. In order to determine the active component of this species, 960 mg of extract was subjected to bioassay-guided fractionation, using the FRAP antioxidant activity assay as a guide throughout the purification process.

A Shimadzu (Columbia, MD, USA) Prominence HPLC system, consisting of a photodiode array (PDA) detector, fraction collector and LC Solution software, was used for all HPLC separations and analyses. For preparative HPLC, a Restek (Bellefonte, PA, USA) 250 × 21.2 mm, 5 μm Ultra II reverse-phase C-18 column was used, employing gradient elution from 20% MeOH:H_2_O to 100% MeOH in 30 min, and then 90 min of elution with 100% MeOH. Flow rate was set to 5 mL/min. For analytical HPLC, a Restek 150 × 4.6 mm, 5 μm Ultra II reverse-phase C-18 column was used, employing gradient elution from 5% MeOH:H_2_O to 100% MeOH in 20 min, and then 40 min of elution with 100% MeOH. Injection volume was 10 μL, flow rate of 1 mL/min, and a controlled column oven temperature of 25 °C.

For the identification of the active principles various analytical methods were used, and compared to spectral data reported in the literature. ^1^H NMR (nuclear magnetic resonance) and ^13^C NMR spectra were recorded in CD_3_OD (Cambridge Isotopes Laboratories, Andover, MA, USA) with a Bruker (Billerica, MA, USA) Avance 400 MHz NMR spectrometer. Electrospray ionization mass spectrometry (ESI-MS) was performed on a Varian (Agilent Technologies, Santa Clara, CA, USA) 500-MS IT mass spectrometer. High-resolution MS was measured with an Agilent Technologies 6530 Accurate-Mass Q-TOF LC/MS. UV/Vis spectral data were taken from the PDA information part of the HPLC recording system (see above).

A reference sample of fucoxanthin (95%, Product No. F6932) was purchased from the Sigma-Aldrich Corp. (St. Louis, MO, USA). The identity of this reference sample was verified by ^1^H NMR and MS as an authentic sample of fucoxanthin employing our routine method for testing of sample purity and authenticity.

### 4.6. Statistics

All data are expressed as means ± SD. Data were analyzed using one-way analysis of variance (ANOVA) tests, as well as *post hoc* tests. A significant difference was considered at the level of *P* < 0.05.

## 5. Conclusions

The results presented in this study represent the first significant assessment of the antioxidant activity of Hawaiian algae. Using the FRAP assay, it was determined that all 30 species of Hawaiian algae tested showed antioxidant activity, and of these the brown algae were statistically the most active. Certain individual species, especially *Turbinaria ornata*, *Gayralia oxysperma*, and *Chaetomorpha antennina*, were found to have significant antioxidant activity. *T. ornata* exhibited the highest overall antioxidant activity, with the carotenoid fucoxanthin being identified as the major component responsible for the majority of this activity. It is apparent from the findings of this study that further investigations are required to assess many of the issues raised in this investigation relating to time and location of collection, as well as, to do with long-term sample storage. Moreover, detailed studies need to be undertaken with individual species that consider the effects of herbivory, light levels, depth, salinity, nutrient levels, type, age, and reproductive stage on observed antioxidant activity. Thus, the antioxidant activity of Hawaiian algae could become the subject of a large intraspecific variation study. Additionally, future studies need to investigate the use of standardized methodologies to enable result comparisons with other studies. This will be a challenge as there are very few if any accepted norms for this type of research. Despite these challenges, the present study stands as the first extensive screening of antioxidant activity of Hawaiian algae, and sets the stage for further research, including the isolation of the antioxidant components present in the most active of the tested algae.
